# “How old do you feel?” Subjective age as a tool to enhance communication in primary care: a pilot observational study

**DOI:** 10.1186/s12875-025-03146-9

**Published:** 2025-12-27

**Authors:** Robert D. Hoffman, Galia Zacay, Joseph Azuri, Tal Hakmon Aronson, Lilach Eyal Waldman, Tanju Yilmazer, Limor Adler

**Affiliations:** 1 Department of Family Medicine, Gray Faculty of Medical & Health Sciences, Tel Aviv University, Tel Aviv, Israel; 2 Department of Family Medicine, Maccabi Healthcare Services, Tel Aviv, Israel; 3 Meuhedet Healthcare Services, Tel Aviv, Israel; 4 Izmir Gazi Hospital Home Care Services, Izmir, Turkey

**Keywords:** Subjective age, Chronological age, Primary care, Communication

## Abstract

**Background:**

Subjective age has been linked to health outcomes, including functional decline and increased mortality risk. However, its utility in primary care settings remains underexplored. This study aimed to evaluate whether the question “How old do you feel?” could enhance physician-patient communication.

**Methods:**

In this pilot observational study, thirteen primary care physicians (PCPs) asked 194 patients aged 50 and older the question “How old do you feel?” during clinical encounters. Following each encounter, PCPs completed a questionnaire to assess the impact of the question on the consultation. Data were analyzed using descriptive statistics, and univariate analysis was performed with a mixed-effects logistic regression model to identify factors influencing physicians’ perceptions of the question’s utility. We also evaluated their free-text responses to understand their perceptions of this question.

**Results:**

PCPs reported positive experiences, with 74% of consultations leading to further discussions, and 80% of PCPs felt the question improved their understanding of the patient. 62% believed the question benefited the patient. Factors such as physician age, experience, and patient multimorbidity were significantly associated with positive perceptions of the question’s utility.

**Discussion:**

This study suggests that asking “How old do you feel?” can enhance communication between PCPs and patients, providing valuable insights into patients’ overall well-being. This question may help identify vulnerable patients and improve care. Future studies should explore its broader applicability and long-term effects in diverse primary care settings to assess its potential as a standard tool in clinical practice.

**Supplementary Information:**

The online version contains supplementary material available at 10.1186/s12875-025-03146-9.

## Background

The concept of subjective age—how old a person feels compared to their chronological age—has been widely studied, particularly in geriatric medicine. Research consistently shows that individuals who feel younger than their actual age tend to have better functional health, greater psychological well-being, and improved health outcomes [1, 2]. Conversely, those who feel older than their chronological age are more likely to experience increased morbidity, cognitive decline, and even higher mortality risk [3, 4]. Several studies suggest that socioeconomic status significantly influences subjective age, with individuals from lower socioeconomic backgrounds more likely to feel older than their actual age due to increased life stressors and poorer health conditions [5].

A 2020 doctoral study by Dr. Tanju Yilmazer explored factors affecting perceived age among 228 individuals and found that being socially active was the strongest predictor of feeling younger [6]. Similarly, broader studies, such as the Midlife Development in the United States (MIDUS) study, have shown a consistent pattern: younger adults (ages 25–29) perceive their age accurately, while older adults increasingly report feeling younger, with the oldest participants (70+) feeling, on average, 13 years younger than their actual age [7, 8]. While psychological factors have been found to contribute to subjective age, they account for only a small but significant portion, with up to 80% of subjective age variation remaining unexplained, highlighting its complexity [9].

Physicians often assume that objective health status plays a central role in how old a patient feels. However, a study by Choi et al. found that objective health indicators were significantly associated with subjective age discrepancy only in individuals aged 70–79, suggesting that subjective age is influenced by more than just medical conditions [10]. Despite this, subjective age has been shown to rival or even outperform chronological age as a predictor of psychological and health-related outcomes [11, 12]. Moreover, interventional studies suggest that modifying perceptions of age can have a positive impact on cognitive function, physical health, and mental well-being [13–15].

Despite the growing evidence linking subjective age to meaningful health and psychological outcomes, its use as a conversational tool in clinical practice has received little attention. Asking a patient, “How old do you feel?” may create an opening for richer communication by inviting patients to share their perceptions of their physical, emotional, and functional well-being in their own words. In primary care—where time is limited, and physicians often rely on brief cues to assess patient concerns—this question may help uncover issues that are not captured by standard medical history-taking, such as fatigue, loneliness, reduced resilience, or social stressors. To our knowledge, no studies to date have formally evaluated the question as a communication tool in routine primary care consultations.

This pilot study aims to assess whether primary care physicians (PCPs) find this question valuable and whether they believe it benefits their patients. We hypothesize that PCPs will find the question useful in their clinical encounter and that its perceived utility will be influenced by both physician characteristics (such as age and gender) and patient characteristics (such as age, gender, and comorbidities). By examining PCPs’ attitudes toward this question, this study represents a first step in evaluating whether subjective age assessment has a role in primary care and whether it could enhance physician-patient communication and overall patient care.

## Methods

### Study design and setting

This exploratory, pilot observational study, using a questionnaire-based data collection approach, examined the use of the question “How old do you feel?” during routine primary care visits with patients aged 50 years and older. Participating PCPs were instructed to ask this question in eligible encounters and complete a brief, anonymized web-based questionnaire (via Google Forms) immediately afterward to reflect on the perceived impact. Physicians were instructed to ask the question after the initial complaint was clarified, towards the end of the consultation.

Each PCP was asked to repeat the process with at least 15 consecutive eligible patients, unless exclusion criteria applied. Patients who declined participation were not counted toward the target. PCPs selected their own start date within the one-month study period. The number of 15 consultations per physician was selected for pragmatic reasons, consistent with the pilot and exploratory nature of the study. This target was considered feasible to complete within a typical single working day in primary care, while still providing sufficient initial data to assess the acceptability and practicality of integrating the question into routine consultations. The goal was to collect at least 100 completed patient questionnaires across all participants.

At the end of the recruitment period, each physician also completed a final questionnaire summarizing their experience. All data were anonymized, entered into an Excel database, and analyzed. The study was approved by the ethical committee of Maccabi Healthcare Services (#MHS-0068-23).

### Participants

Eligible participants were patients aged 50 years or older attending routine primary care visits. Exclusion criteria included patients with significant language barriers or other factors that precluded meaningful participation. The inclusion criteria for PCPs were working at Maccabi Healthcare Services (MHS), board certification in family medicine, active involvement in resident training, and maintaining an active clinical practice. MHS is the second largest healthcare fund in Israel, insuring over 2.8 million nationwide.

### Survey instrument

The study’s lead authors developed the questionnaire after a literature search revealed no comparable tools. There was a discussion about the study aims and potential factors influencing clinicians’ responses to the subjective age question. Several rounds of email feedback were conducted among the study team to establish face and content validity. The questionnaire was then pilot-tested on 4 PCPs, and participant feedback was used to refine and finalize the instrument. Particular attention was given to keeping the questionnaire brief and easy to complete, as it needed to be filled out during routine clinical visits.

The encounter-specific questionnaire included the main question (“How old do you feel?”), basic patient demographics, and the physician’s immediate perception of the question’s impact on the encounter (the questionnaire is presented in the supplementary material).

At the end of the study, a separate final questionnaire was administered to collect demographic information on the PCPs, including both open- and closed-ended questions about their overall impressions of integrating the subjective age question into clinical practice. Free-text responses were included in the analysis to complement the quantitative findings.

### Statistical analysis

We defined age difference as the actual age minus the subjective age. Descriptive statistics were used to summarize the characteristics of participating PCPs and patients. Univariate analysis was conducted using chi-square tests for categorical variables and Student’s t-test or Mann-Whitney U test for continuous variables.

Univariate analysis was performed using mixed effects logistic regressions model to identify factors associated with PCPs finding the question useful while controlling for the physician’s identity. The analysis was performed with a single fixed effect, i.e., one of the physician’s characteristics or the patient’s characteristics. We used R version 4.1.0 (R Foundation for Statistical Computing) for all statistical analyses.

## Results

### Participants

Thirteen PCPs from MHS participated in the study during February 2024. Each physician offered participation to at least 15 consecutive eligible patients, for a total of 208 patients approached.

### Physician demographics and personal subjective age perception

The participating physicians’ demographics are summarized in Table 1. The age range was 39–65 years, with seven physicians under age 50. There were 7 women and 6 men. All physicians worked in an urban setting, and 6 (46.2%) described their clinic workload as heavy or very heavy.

**Table 1:** Primary care physicians’ characteristics

Physicians	*N* = 13 Number (percentage)
Sex: females	7 (53.8)
Age:	
Real age med [IQR]	46 [41–52]
Subjective age med [IQR]	40 [35–46]
Age-difference (real – subjective), med [IQR]	9 [0–10]
Workload at the clinic:	
Light	1 (7.7)
Moderate	6 (46.2)
Heavy	5 (38.5)
Very heavy	1 (7.7)

Most physicians perceived their own age as younger than their chronological age (range 0–15 years younger). Four out of 13 reported no age difference, while six reported feeling up to 10 years younger than their chronological age.

### Patients’ characteristics

Of the 208 eligible patients according to the study protocol, 13 were not invited to participate, and one did not understand the question. The reasons for not inviting these patients included time constraints (*n* = 5), feeling that the question was inappropriate (*n* = 6), and feeling uncomfortable with patient-physician interaction (*n* = 2).

The demographics of the 194 participating patients are summarized in Table 2. Ninety-four patients were younger than 65 years old, 101 (52%) felt that they were younger by 10 years or more than their actual age, while 27 (14%) felt older than their actual age. 58% (113) were female, 55% were working, 34% were retired, and most of them were married (78%). Only 33 patients (17%) were healthy without any illness or disability.

**Table 2:** Patients’ characteristics

	*N* (%)
N	194
Sex: females	113 (58.2)
Age:	
Real age med [IQR]	66 [57–72]
Subjective age med [IQR]	53 [45–65]
Age-difference (real – subjective) med [IQR]	10 [0.25–19]
Marital status:	
Married or in a relationship	152 (78.4)
Single	6 (3.1)
Divorced	21 (10.8)
Widow	15 (7.7)
Employment:	
Employed	107 (55.2)
Retired	66 (34.0)
Unemployed	21 (10.8)
PCP perceived Health status:	
Healthy	33 (17.0)
Mild illness/disability	91 (46.9)
Moderate illness/disability	60 (30.9)
Severe illness/disability	10 (5.2)

### PCPs’ perception of utilizing the research question

Most physicians had a positive experience with the question; in 80.4% of encounters, they felt it aided their understanding of the patient, though in only 44.8% of encounters, they believed it directly helped in the current encounter. In 63.9% of encounters, physicians felt that asking the question benefited the patient. Furthermore, in 76.8% of encounters, the question led to a discussion with the patient. More than half of the patients’ responses (55.2%) surprised the physicians. The full responses are presented in Tables 3 and 4.

**Table 3:** Responses to the questionnaire

How helpful was this question in understanding the patient better?									
1 (not at all)	2			3		4			5 (very helpful)
21 (10.8%)	17 (8.8%)			74 (38.1%)		52 (26.8%)			30 (15.5%)
How helpful was this question to the current clinical visit?									
1 (not at all)	2			3		4			5 (very helpful)
69 (35.6%)	38 (19.6%)			44 (22.7%)		32 (16.5%)			11 (5.7%)
Did the question lead to a conversation with the patient?									
No					Yes				
45 (23.2%)					149 (76.8%)				
Do you feel the patient benefited from you asking the question?									
No					Yes				
70 (36.1%)					124 (63.9%)				
How do you think the patient reacted to the question?									
Positively		Neutrally			Negatively			Undecided	
129 (66.5%)		56 (28.9%)			7 (3.6%)			2 (1.0%)	
Were you surprised by the patient’s answer?									
No			Yes				Undecided		
86 (44.3%)			107 (55.2%)				1 (0.5%)		
Was asking the question “worth” the time spent?									
No			Yes				Undecided		
49 (25.3%)			144 (74.2%)				1 (0.5%)		

**Table 4:** Participants’ responses to the open-ended questionnaire item

• The question created a positive atmosphere, with a smile. It lowered tension. • This is a tool to understand how much the totality is a burden on the patient. I was positively surprised to discover their feelings in some of the patients. They feel young despite significant illnesses. The question sometimes takes time as it leads to a discussion. I think it is useful as a tool to start a conversation • Although many of my answers were that the question didn’t teach me much, it was interesting! And it was always accepted with a positive attitude. • Very positive. Sometimes leads to surprising personal revelations and a deeper understanding of the patient’s health and life. • Positive - nice idea • It was certainly an interesting experience, but I do not think I will use it • The answers were interesting and taught us how the patients see themselves, often to my surprise. It does not add much time to the visit and brings a smile to most patients’ faces. • I was surprised by the patients’ answer and the benefits of this simple question • I think this question can make patients feel better about themselves and their medical condition. • I like the question. • Time-consuming • Helpful with understanding the patient’s well-being in a very short time • I continued asking even after filling the quota • On the other hand, it does extend the visit, not necessarily efficiently. • I consider using it routinely • I intend to use it in my clinic • It was fun! Thank you!	

There was an association between the physician’s view of the question as helpful and the physician’s age difference, with an odds ratio (OR) of 1.1 for each additional year (confidence interval [CI] 1.002–1.21, *p* < 0.05). No association was found with the physician’s age, gender, or workload at the clinic. Similarly, no association was found between the physician’s view of the question as helpful and the patient’s age, age difference, gender, or other characteristics.

Multivariate analysis was not performed due to the lack of associations found in univariate analysis.

### Physicians’ reflections

Many free-text responses from PCPs expressed a positive experience and a willingness to use the question in future consultations. One physician noted: “*The question created a positive atmosphere*,* with a smile*,* and lessened tension*.” (Male, age 54). Another physician commented: “*… I was positively surprised to discover the feelings of some of the patients. They feel young despite significant illnesses. The question sometimes takes time as it leads to a discussion. I think it is useful as a tool to start a conversation.*” (Female age 42). Another (Female, age 44) noted: “*Helpful with understanding the patient’s well-being in a very short time.*”

One physician commented: “*It was certainly an interesting experience*,* but I don’t think I will use it.*” (Female, age 52).

PCPs commented they felt this question was personal: “*asking a personal question*,” while another noted that it “*sometimes leads to surprising personal revelations and a deeper understanding of the patient’s health and life.*” (Male, age 65).

## Discussion

### Main findings

This study explored the utility of asking patients the question “How old do you feel?” in primary care consultations. Our findings indicated that most PCPs found this question useful in fostering discussion and deepening their understanding of patients. Many PCPs reported being surprised by their patients’ responses, and in 74% of consultations, the question led to further dialogue. Additionally, physicians found that in 80% of cases, the question helped them understand their patients better, and 62% believed it benefited the patient. Free-text responses from PCPs highlighted the positive impact of the question on patient engagement and rapport-building.

### Interpretation

The question “How old do you feel?” appears to be a concise and meaningful tool for capturing patients’ perceptions of their overall health and well-being. It invites individuals to integrate various aspects of their physical and mental health into a single numerical value, potentially uncovering unexpected insights that may not be captured through standard clinical assessments. Importantly, by shifting the focus from biomedical parameters to the patient’s subjective experience, the question may enhance doctor–patient communication. It can open space for patients to articulate concerns about fatigue, mood, resilience, or functional decline in a way that feels natural and non-threatening, thereby strengthening rapport and facilitating more person-centered conversations [16–19]. In the fast-paced environment of primary care, where time constraints often limit deeper discussion, this question may serve as an efficient entry point into topics that would otherwise go unaddressed.

Subjective age has been extensively studied and has been associated with multiple health outcomes, including hospitalization outcomes [20], mental, cognitive, and physical functioning [21], frailty [22], risk for falling [23], stroke, and cardiovascular diseases [24, 25]. Moreover, prior research has shown that feeling older than one’s chronological age predicts higher mortality risk [26, 27], while feeling younger is linked to increased longevity [15]. Given these associations, subjective age may provide primary care physicians with meaningful patient-reported information that can help identify individuals at higher risk for injury, functional decline, or mortality, thereby prompting timely evaluation or preventive intervention [15, 28].

One concern in primary care is that such a question might label a large proportion of patients as “high risk.” While 14% may be considered a relatively high proportion in a primary care setting, this finding is consistent with population-based data showing that 8–10% of adults report feeling older than their chronological age [28]. Rather than over-identifying patients, this question appears to highlight a substantial, clinically meaningful subgroup that may benefit from further inquiry into functional status, mood, or overall well-being.

Subjective age can be modified through targeted interventions. Research suggests that physical therapy, exercise programs, balance training, and health messaging promoting positive attitudes toward aging can improve subjective age perception [13, 15, 29]. This modifiability enhances its potential clinical value, offering both diagnostic insight and a pathway for intervention.

Some physicians reported that they refrained from asking the question in a subset of encounters because they felt it was inappropriate or that the patient might be uncomfortable. This hesitation highlights potential barriers to integrating the question into routine consultations, including concerns about patient vulnerability, contextual fit, and the physician’s own comfort level. Future research is needed to further explore these barriers and to understand the conditions under which subjective-age questions can be used effectively and sensitively in primary care.

### Implications

The addition of this question to the primary care “tool chest” may be of great benefit to PCPs. Although PCP follow their patients through health and sickness, sometimes over decades, it is an uncommon event that they inquire about their patients’ subjective perception of their health status. By asking a simple but unexpected question, the patient provides an immediate and intuitive response that may reflect deep perception and beliefs. PCPs can identify patients at higher risk and focus on improving their health in areas that they find important.

### Strengths and limitations

This study offers preliminary insights into PCPs’ perceptions of the utility of subjective age assessment in primary care. The positive responses suggest that this question has the potential to enhance patient-provider communication and deepen physicians’ understanding of their patients’ well-being.

However, several limitations should be noted. First, the questionnaire used in this study was developed specifically for this project and has not undergone formal psychometric validation. As a result, responses to the unvalidated Likert-type items should be interpreted with caution, and the findings considered exploratory rather than definitive. Second, the study was conducted with a small sample of PCPs from a single healthcare organization, limiting the generalizability of the findings. Third, we were unable to identify specific factors that influenced PCPs’ perceptions of the question’s usefulness, which could be explored in future research. Fourth, this pilot study examined only the clinician perspective and did not include patient input in the design or evaluation of the question. Given the inherently patient-centered nature of subjective age, future studies should incorporate patient voices to better understand how they experience and interpret this question. Qualitative methods can enrich our understanding of the topic.

## Conclusion

Integrating the question “How old do you feel?” into primary care consultations appears to be a promising addition for enhancing physician-patient communication and identifying patients at potential health risk. The positive responses from participating PCPs suggest that this simple question facilitated meaningful dialogue, improved physicians’ understanding of patients, and may serve as a tool for the early identification of vulnerable individuals. Future studies should assess its effectiveness in diverse primary care settings. Expanding research in this area could lead to the incorporation of subjective age assessment into standard primary care practice, ultimately benefiting both physicians and patients.

## Supplementary Information

Supplementary Material 1.

## Data Availability

The datasets used and/or analysed during the current study are available from the corresponding author on reasonable request.

## Abbreviations

MHS: Maccabi Healthcare Services
OR: Odds ratio
PCPs: Primary care physicians
