# Supplementary material for: “How old do you feel?” Subjective age as a tool to enhance communication in primary care: a pilot observational study
Source: BMC Prim Care. 2025 Dec 27;27:27. doi: 10.1186/s12875-025-03146-9 (PMC12853589; doi:10.1186/s12875-025-03146-9)
Supplement: Supplementary file 1 — Supplementary Material 1. [file 12875_2025_3146_MOESM1_ESM.docx]

**The Questionnaire:**

**Patients’ demographics**

**1. Age** ___

**2. Gender** *M / F / Non Binary*

**3. Employment:***working / disabled and not working/ retired*

**4. Marital status**: *Single / Married/ Divorced*

**5. General health:**

*0 healthy*

*1 mild illness/disability*

*2 moderate illnesses/disability*

*3 severe illnesses/disability*

** If patient skipped – what was the main reason?

1. Time pressure in the clinic?
2. Uncomfortable with patient–physician interaction?
3. Not appropriate with the current medical issue?
4. Other: ___________________________________

**"HOW OLD DO YOU FEEL?"**

**How old did the patient feel? ___**

**1.   How helpful was this question to understanding more about the patient?** 1 2 3 4 5 (5 very)

**2.  How helpful was this question to the current clinical visit?**

1 2 3 4 5 (5 very)

**3. Did the question lead to a conversation with the patient?** *Yes / no*

**4. Do you feel the patient benefited from you asking the question?** *Yes / no*

**5. How do you think the patient reacted to the question?** *Negatively/neutral/positively*

**6. Were you surprised by the patient’s answer?** *Yes /No*

7. **Was asking the question "worth" the time spent?** *Yes/No*

**GP demographics**

**1. Age**

**2. Gender**

**3. Years since received MD**(*up to 5 / up to 10 / up to 15 / up to 20 / more than 20*)

**4. Clinical workload (self-estimate of load)** *1 very light 2 light 3 moderate 4 heavy 5 very heavy*

**5. Clinical setting** (*urban/rural/mixed*)

**6. How old do you feel?**

**7. What were your thoughts about using this question as part of your patient consultations?** *(open question)*
